# An optimized imaging protocol for orofacial cleft patients

**DOI:** 10.1002/cre2.123

**Published:** 2018-07-25

**Authors:** Dries De Mulder, Maria Cadenas de Llano‐Pérula, Guy Willems, Reinhilde Jacobs, Jakob Titiaan Dormaar, Anna Verdonck

**Affiliations:** ^1^ Department of Oral Health Sciences‐Orthodontics, KU Leuven and Dentistry University Hospitals Leuven Belgium; ^2^ OMFS IMPATH, Department of Imaging & Pathology, Faculty of Medicine, University Leuven and Oral and Maxillofacial Surgery University Hospitals Leuven Belgium; ^3^ Department of Dental Medicine Karolinska Institutet Sweden

**Keywords:** cleft lip and palate, cone beam computed tomography, imaging, orofacial cleft, radiological guidelines

## Abstract

The objective was to present an optimized imaging protocol for orofacial cleft (OFC) patients, which might be used as an international recommendation for OFC care programs. The present radiological protocol has been structured by the OFC team of the University Hospitals Leuven based on a combined approach of clinical experience and scientific evidence. The development was based on careful monitoring of the existing needs for radiological diagnosis by the involved disciplines. Needs were revised by expert consensus and radiological optimization. Effective doses were converted to panoramic equivalents (professional conversion) and background radiation (patient conversion). At the age of 6, a panoramic radiograph is taken for the evaluation of dental anomalies. For the preoperative planning of secondary alveolar bone, grafting a low‐resolution cone beam computer tomography (CBCT) of a limited field of the maxilla is taken at the age of 7 to 9. At the age of 10, 15, and 20, a low‐resolution CBCT of both jaws with the smallest possible field is taken serving as conventional, presurgical, and end of treatment records, respectively. Two‐dimensional images are reconstructed out of 3D ones. There are currently no international guidelines concerning the imaging protocol for OFC patients. It is clear that a multidisciplinary approach plays a key role in radiation hygiene. In this article, we presented an optimized imaging protocol for OFC patients based on European guidelines to accomplish the concepts of justification and optimization, which might be used as an international recommendation for OFC care programs.

## 1 INTRODUCTION

Orofacial clefts (OFC) are common congenital malformations of the lip and/or palate, caused by a complex interaction of genetic and environmental factors (Wehby & Murray, 2010). OFC patients often deal with speech, masticatory and hearing problems, dental and craniofacial anomalies, and psychosocial issues. Given the complexity of the pathological conditions in these patients, a multidisciplinary treatment approach is of outmost importance. Therefore, OFC teams may typically organize multidisciplinary consultations approximately once a year for each patient to accommodate treatment planning across different disciplines such as otorhinolaryngology, maxillofacial surgery, orthodontics, general dentistry, radiology, human genetics, psychology, speech therapy, and social work. OFC care programs normally start at the first week of life and ends up in adulthood (Auslander et al., 1993; Shaw, Semb, Nelson, Brattstrom, & Molsted, 2001).

One important aspect in the OFC care program is diagnostic imaging. Given the multidisciplinary setting, different radiological projections may be required for diagnostic, presurgical, and postoperative assessment by different specialties throughout the lifespan of an OFC patient. The latter may lead to cumulative radiation throughout childhood and adolescence that means an increased radiation risk (Jacobs et al., 2017; Pauwels et al., 2014a).

Because of this, OFC protocols should strive for optimized imaging during the various treatment phases up until adulthood. Surprisingly, there are no international guidelines in literature concerning such longitudinal imaging protocol for OFC patients.

The aim of this article is to present an optimized imaging protocol for OFC patients, which might be used as an international recommendation for OFC care programs. Subobjectives include justification of the required imaging steps and optimization of the related radiation doses versus required image quality at various time points throughout the entire treatment of the OFC patient.

## 2 MATERIALS AND METHODS

The present imaging protocol has been structured by the OFC team of the University Hospitals Leuven, based on a combined approach of clinical experience and scientific evidence.

According to the European guidelines for OFC patients (Shaw, Semb, Nelson, Brattstrom, & Molsted, 2001), clinical records (radiographs, study casts, intraoral, and extraoral photographs) should be taken for treatment planning, monitoring, and evaluation. Records are collected at the age of 6 (start of treatment), 10 (conventional treatment planning), 15 (presurgical records), and 20 (end of the craniofacial growth). The development of this standardized imaging protocol was based on careful monitoring of the existing needs for radiological diagnosis by the different specialists involved in the team. Needs were revised by expert consensus and radiological optimization. A first action (expert consensus) consisted of moving the necessary time points indicated by the different specialties, up until a level where these would better overlap. The second action (radiological optimization) aimed to optimize the imaging characteristics and the radiological protocol adapted to the diagnostic needs for each specific time point. This optimization included resolution (high resolution = HR or low resolution = LR), nature of the image (two‐dimensional = 2D or three‐dimensional = 3D), and anatomical field of view (FOV). After critical appraisal, the timing and settings of the needed radiographs were carefully adjusted to accomplish the concepts of optimization and justification.

Instead of emphasizing the absolute value of the effective dose ranges, we converted them to panoramic equivalents for professionals and background radiation for patients (Oenning et al., 2017). For this conversion, we used 10 μSv for one panoramic radiograph and 5,1 mSv background radiation per year (Bornstein, Scarfe, Vaughn, & Jacobs, 2014).

## 3 RESULTS

Optimization included a reduction in the number of images, selection of low‐dose imaging techniques, and limiting the required FOV as much as possible. Table 1 provides a summary of the present optimized imaging protocol for OFC patients. Table 2 represents the effective dose ranges of the discussed imaging steps converted to panoramic equivalents and background radiation.

**Table 1:** Optimized imaging protocol for OFC patients

Number	Age (years)	Image type	Resolution	FOV (cm^2^)	Justification
1	6	Pano			Evaluation of dental anomalies
					Impaction
					Agenesis
					Supernumerary teeth
					Caries
					Position maxillary lateral incisor
2	7–9	CBCT	LR	Maxilla (8 × 5)	Preop SABG planning
					Cleft size, shape, and volume
					Relationship with anatomical structures
3	10	CBCT	LR	Both jaws, including N, C, and S (12 × 15)	Conventional orthodontic treatment records
			(*) HR	(*) maxillary canine region (smallest possible FOV)	Evaluation of
					Bone bridge
					Dental and craniofacial development
					Condition of cleft adjacent teeth
					Canine eruption
					Caries
					Canine impaction (*)
4	15	CBCT	LR	Both jaws, including N, C, and S (12 × 15)	Presurgical records
					Evaluation of
					Extent of skeletal discrepancy
					Relation M3 to alveolar nerve
					Residual opening: TABG?
					Caries
5	20	CBCT	LR	Both jaws, including N, C, and S (12 × 15)	End of treatment records
					Preimplant planning?
					M3 extraction?

*Note*. Pano: panoramic radiograph; CBCT: cone beam computer tomography; LR: low resolution; HR: high resolution; FOV: field of view; N: nasion; C: chin; S: sella turcica; SABG: secondary alveolar bone grafting; TABG: tertiary alveolar bone grafting; M3: third molar.

**Table 2:** Effective dose conversion from the images of Table 1

#	Effective dose range (μSv)	Professional conversion: Panoramic equivalents	Patient conversion: Background radiation
1	6–10	1	10–17 hr
2	43–63	4–6	3–4.5 days
3	81–216	8–22	6–15 days
	(*) 16–33	1.5–3	1–2 days
4	81–216	8–22	6–15 days
5	81–216	8–22	6–15 days

Generally, before the age of 6, treatment of OFC patients consists of clinical interventions that do not require any radiological projections.

At the age of 6, a panoramic radiograph is taken to evaluate dental anomalies such as impaction, missing or supernumerary teeth, caries, and the position of the maxillary lateral incisor. The planning of secondary alveolar bone grafting (SABG) depends on the position of the maxillary lateral incisor. If it is located in the premaxilla, the bone graft is performed in function of the root formation of the maxillary canine. If the maxillary lateral incisor tends to erupt in the lateral segment, an early SABG is planned. In case of more complex pathologies and syndromes involving craniofacial structures (e.g., hemifacial microsomia, Pierre Robin sequence, craniosynostosis, and early childhood caries), a panoramic or 3D radiograph is taken at an earlier age (Anderson, Yong, Surman, Rajion, & Ranjitkar, 2014).

**Figure 1:**
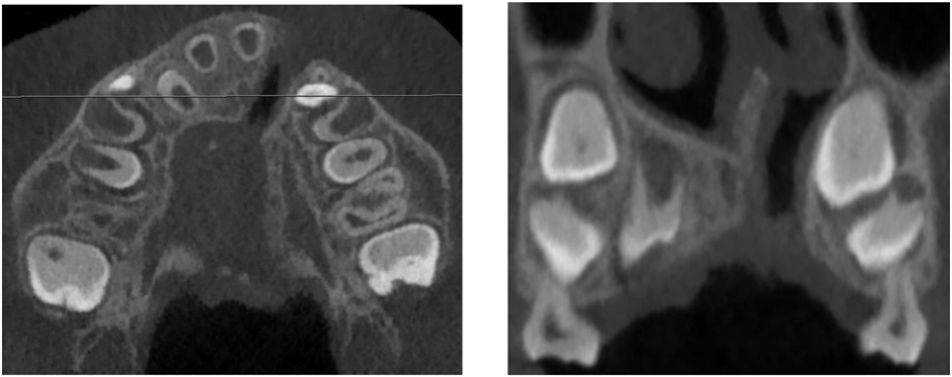
Low‐resolution CBCT of the maxilla with FOV 8 × 5 cm^2^ in an 8‐year‐old patient with unilateral cleft lip and palate for preoperative SABG planning. Axial slice (left) with the corresponding coronal slice (right) indicated by the line on the axial slice

At 7 to 9 years old, SABG needs to be performed to restore the alveolar defect, permitting canine eruption through the bone graft. This is ideally planned when the root of the unerupted canine is a half to two‐thirds developed (Oh, Park, Choi, Kwon, & Koh, 2015). For preoperative planning of SABG, a low‐resolution CBCT of the maxilla is taken (Figure 1). The limited FOV (generally 8 × 5 cm^2^) minimizes radiation exposure to radiosensitive structures such as the eye lens and the thyroid gland. Three‐dimensional evaluation of the alveolar cleft enables insight in size, shape, and volume of the cleft and its relationship with anatomical structures, thereby increasing the procedure's predictability (Choi et al., 2012). It has been shown that low‐dose protocols are sufficient to achieve these goals (Oenning et al., 2017). Postoperatively, patients are regularly seen by the maxillofacial surgeon to evaluate the healing and integration of the bone graft. No further radiographs are needed in this time frame.

**Figure 2:**
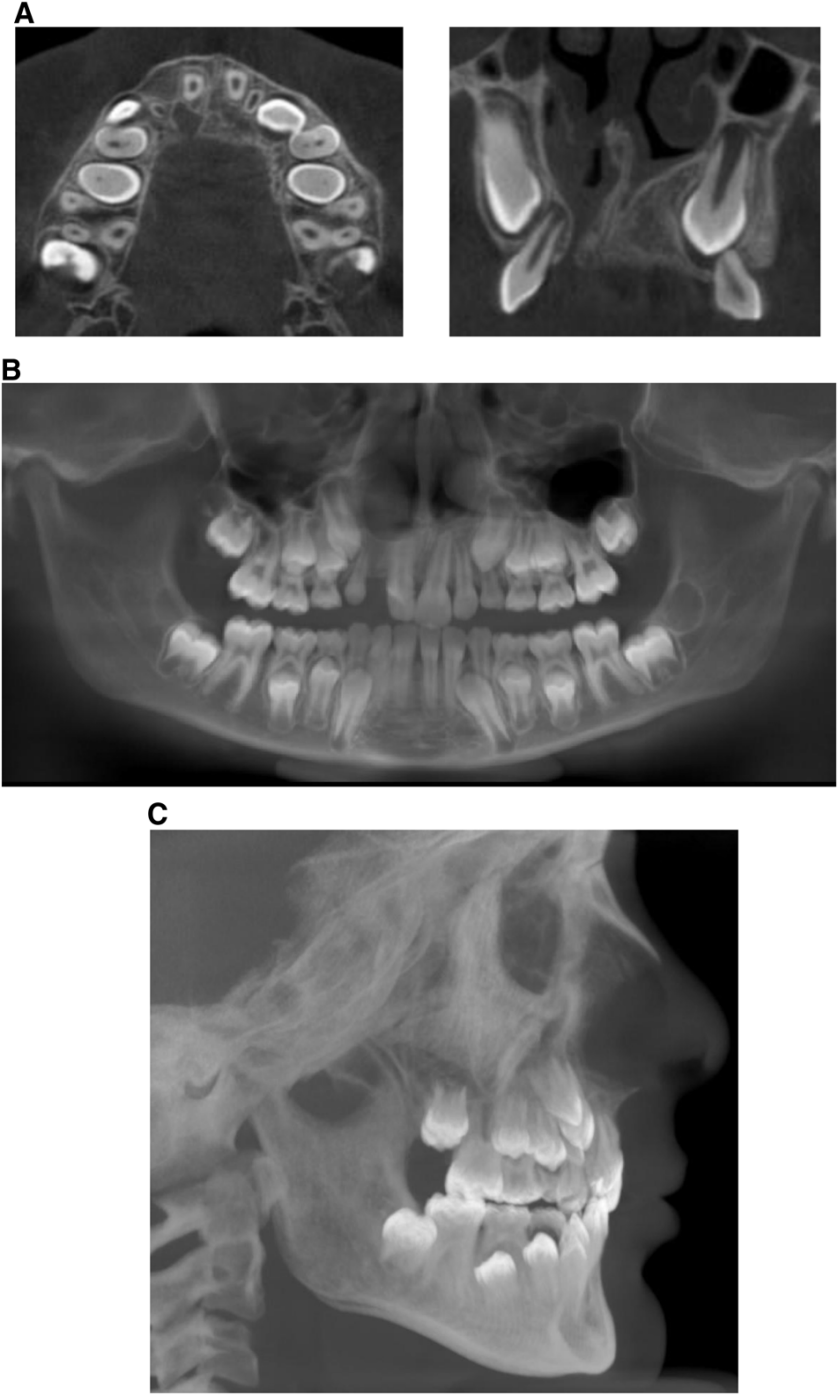
(A) Low‐resolution CBCT of both jaws with FOV 12 × 15 cm^2^ in a 10‐year‐old patient with unilateral cleft lip and palate before the start of the conventional orthodontic treatment. The 3D position and relation of the cleft‐side lateral incisor and canine erupting through the bone graft can be accurately assessed on the axial (left) and coronal (right) slice. (B) Panoramic reconstruction of the low‐resolution CBCT of both jaws depicted in Figure 2A. (C) Cephalometric reconstruction of a low‐resolution CBCT of both jaws

At the age of 10, records are collected prior to the conventional orthodontic treatment. The timing of this treatment stage depends on dental development, more specifically the eruption of the cleft‐side maxillary canine through the bone graft. A low‐resolution CBCT of both jaws with a FOV of generally 12 × 15 cm^2^ is taken (Figure 2a), where canine eruption and the status of the bone bridge are evaluated. Important cephalometric landmarks (nasion, sella turcica, and the chin) should be included in the FOV, enabling cephalometric analysis. Redundant radiation is avoided by reconstructing a panoramic radiograph (Figure 2b) and a lateral cephalogram (Figure 2c) out of the CBCT image. The orthodontist can then evaluate dental and craniofacial development in order to set up the orthodontic treatment plan, while the pediatric dentist can perform accurate caries assessment. Low‐dose protocols are sufficient for these tasks, unless detailed evaluation of canine impaction is required. In that case, a high‐resolution 3D image is needed because a low‐resolution image cannot fulfill the diagnostic requirements for canine impaction. A high‐resolution image of a limited field of the maxilla (canine and adjacent teeth only) is justified as 3D imaging can significantly impact the treatment approach (European Commission, 2012).

At the age of 15, presurgical records are collected. A deviation of 1 year is acceptable for this image, because it should be related to the clinical need of the specific case. A low‐resolution CBCT of both jaws with the same settings as described above is taken. Also, a panoramic radiograph and lateral cephalogram are reconstructed out of this 3D image. The extent of the skeletal discrepancy will be decisive for the maxillofacial surgeon and orthodontist to decide upon the need for orthognathic surgery. In case of normal craniofacial growth, orthodontic treatment is the final treatment stage, and these will serve as final records. In case there is skeletal class III due to maxillary growth restriction, orthognathic surgery is indicated once maxillofacial growth has been completed. Residual craniofacial growth is estimated on the reconstructed lateral cephalogram using the cervical stage of maturation method (Gonzalez, 2012). At this stage, 3D images also serve as a diagnostic tool to decide upon the need for third molar removal and to prepare tertiary alveolar bone grafting if a residual opening in the alveolar ridge would exist.

At the age of 20, final treatment records are collected to compare possible relapse during the retention period, aid implant planning, and follow up the third molars. A low‐resolution CBCT of both jaws is taken, including reconstruction of 2D images in analogy with previous images.

## 4 DISCUSSION

### 4.1 Justification

Traditionally, 2D radiographs have been used for diagnostic purposes in OFC teams. Since the introduction of 3D imaging in dentistry, CBCT scans started to gain popularity especially in the field of maxillofacial surgery since the reliance on 2D radiographs is acknowledged to be problematic in presurgical planning and postoperative assessment of alveolar clefts, bone bridges, and cleft‐adjacent teeth as a consequence of trying to derive complex 3D structures from a 2D image. The information derived from 3D images is advantageous over the conventional approach by excluding many factors affecting image quality and reliability, such as enlargement, distortion, structural overlap, and positioning problems. Literature is unanimous that 3D imaging improves diagnosis, treatment planning, and treatment outcomes in OFC patients. Vital diagnostic information such as amount and quality of available bone, complex tooth eruption scheme, or position of impacted teeth cannot be accurately assessed on 2D images (Choi et al., 2012; European Commission, 2012; Jacobs, 2011). Therefore, a higher accuracy of the records can be achieved leading to a higher predictability of interventions by several disciplines.

In the Eurocleft project as well as the Americleft project, recommendations regarding imaging were found to be missing or out of date. We believe that these outdated documents should be updated, and that the present imaging protocol could serve as a guideline.

One could think that 3D imaging implies increased cumulative radiation dose compared with several 2D images. Following the As Low As Reasonably Achievable (ALARA) principle, cumulative radiation dose should be limited through the years, especially in pediatric patients being more sensitive to radiation (Jacobs et al., 2017; Oenning et al., 2017). Complying with this principle for the multidisciplinary treatment approach of OFC patients was the main goal of the present protocol.

Evidence‐based CBCT guidelines for dental and maxillofacial radiology published by the European Commission (SedentexCT) were consulted during the development of this imaging protocol (European Commission, 2012). Although this is a comprehensive document, the guidelines for OFC patients are scarce. Therefore, we implemented the general concepts by applying them on this specific population. According to those guidelines, the use of CBCT is justified for OFC patients as the effective dose is reduced up to 12.3‐fold compared with medical CT (European Commission, 2012; Ludlow & Ivanovic, 2008). On the other hand, frequent CBCT imaging should be avoided unless each exposure can be individually justified. Also, various aspects of radiological protection were included in this imaging protocol in accordance with the basic principles for use of CBCT stated by the European Academy of Dental and Maxillofacial Radiology (European Commission, 2012; Horner, Islam, Flygare, Tsiklakis, & Whaites, 2009).

Effective dose conversion (see Table 2) is a valuable justification tool for professionals and patients to better understand the relevance of absorbed doses. However, broad dose ranges are reported in literature because of differences in patients' age, type of device, and exposure settings.

### 4.2 Optimization

Several image optimization measures were implemented to ensure adequate image quality at the lowest achievable dose (Pauwels, 2015).

First, the overall amount of exposures in the lifespan of an OFC patient was reduced. We restructured the former policy of excessive uncoordinated exposures (both 2D and 3D) by different disciplines leading to high cumulative doses (Jacobs et al., 2017). A massive cumulative dose reduction was obtained by attuning all disciplines with a request for imaging to each other. One carefully selected CBCT image is able to yield all requested information for several disciplines, replacing a cascade of 2D images. Reconstructing 2D images out of a CBCT further optimizes the efficiency of the 3D image.

Secondly, the exposure parameters must be fine‐tuned individually. Reduced exposure parameters such as current (mA), voltage (kV), and exposure time (s) should always be pursued, especially in children. The smallest possible FOV must be chosen because of dose reduction and image quality improvement. The process of scout viewing is required to adapt the FOV to the individual patient. Half‐scan modes (decreased amount of projections), tube current modulation, and tube current‐exposure time product reductions are also encouraged for cleft evaluation in pediatric patients (Oenning et al., 2017; Pauwels et al., 2014b; Stratis et al., 2013). Some devices have automatic exposure control that limits the dose automatically.

Finally, motion artifacts are a recurrent issue as they can negatively influence image quality. An ongoing prospective study in our institution showed that these artifacts are commonly seen in OFC patients, directly related to age and psychological maturity. The older the patients were, the less frequent motion artifacts occurred. Syndromes including mental retardation may also need to be considered when deciding upon a certain radiological projection. Although the scanning device can be adapted to immobilize the patient by providing a stable chair, handgrips, a bite block, a chin, and headrest, the role of the operator should not be underestimated. The operator should have experience with pediatric patients to be able to comfort them during positioning and exposure. In case the FOV should contain the maxilla only, a bite block can be used to improve patient's stability. Bite blocks are not allowed during a CBCT scan of both jaws as disclusion disturbs the reliability of cephalometric measurements. In case there is evidence of motion during scout viewing, it may be better to postpone the radiograph to a later date.

Although an imaging protocol based on chronological ages serves as a backbone, we must keep in mind that every patient should be considered individually. Several factors such as dental age, maturity, cleft type and severity, and other pathologies influence the extent of the treatment strategy as well as the timing and type of radiographs. Also, we realize that an imaging protocol is center‐specific since it depends on the clinical protocol used.

A panoramic radiograph can sometimes be delayed in function of dental maturity. In case of an early SABG, the CBCT for preoperative SABG planning is made earlier. Therefore, standard deviations on the average chronological ages do exist.

The involvement of the alveolar ridge is an important determinant in the imaging protocol. In patients with unilateral cleft lip and palate, bilateral cleft lip and palate, and cleft lip and alveolus, the present imaging protocol is followed. In patients with cleft lip (CL) and cleft palate (CP), conventional 2D radiographs are usually sufficient because bone grafting and orthognathic surgery are indicated less frequently.

## 5 CONCLUSIONS

There are currently no international guidelines concerning the imaging protocol for OFC patients. It is clear that a multidisciplinary approach plays a key role in radiation hygiene. In this article, we presented an optimized imaging protocol for OFC patients based on European guidelines to accomplish the concepts of justification and optimization, which might be used as an international recommendation for OFC care programs.
